# The nonconscious cessation of affiliative motivation: A replication and extension study

**DOI:** 10.1371/journal.pone.0198899

**Published:** 2018-06-28

**Authors:** Stefan Engeser, Birk Hagemeyer, Henk Aarts

**Affiliations:** 1 Department of Psychology, Friedrich Schiller University Jena, Jena, Germany; 2 Department of Psychology, Utrecht University, Utrecht, The Netherlands; Christchurch Hospital, NEW ZEALAND

## Abstract

Previous research has documented that incidentally processed action-words can produce corresponding behavior and that affective-motivational processes modulate these effects. The present study aimed to (1) replicate earlier work showing that behavioral effects of exposure to social affiliation related action-words (e.g., socialize, party, going-out) cease when these action-words are co-activated with negative stimuli, (2) probe moderation effects of individual differences in the affiliation motive, and (3) examine whether action-word priming effects on behavior rely on specific-word associations rather than the activation of a broad concept. Results of an experimental study (*N* = 191) showed that exposure-effects of affiliation related words on behavior instrumental in attaining affiliation goals cease when these words were co-activated with negative affect, but this cessation effect was relatively weak and non-significant. Subsequent analyses revealed that the effect was moderated by the affiliation motive: The cessation effect mainly occurred for individuals with a strong affiliation motive. Further, we found no evidence that word priming effects do merely occur via specific-word associations.

## Introduction

Research in psychological science has a strong fascination and interest in understanding and examining how exposure to semantic information produces corresponding behavioral changes without individuals’ conscious decisions. For example, research on speech production has revealed that the act of speaking rapidly runs through a series of steps, in which the activation of concepts leads to articulation in a rather automatic fashion [1]. Furthermore, studies on the semantic-motor resonance principle suggest that the mere processing of action verbs (e.g., kick) produces corresponding activation in sensory-motor areas, and hence, prepares the motor system to produce the action [2, 3]. Other research suggests that these action-word priming effects might even be extrapolated to more abstract and broader behavioral concepts, such as task achievement or food consumption (e.g. [4, 5, 6]).

Recently, some of these action-word priming effects have been put under scrutiny in an effort for replication. Whereas the replication success rate seems to be rather low so far [7, 8, 9], a recent meta-analysis on word priming effects on behavior revealed a reliable but relatively small (*d* = 0.33) effect [10]. Assuming that the effects are real, and not merely noise, the small effect size indicates that previous word priming effects on behavior are likely overestimated and not easily replicated, because only studies including a relatively high number of participants will have sufficient statistical power to detect it. Additionally, the meta-analysis disclosed a few moderators of action-word priming effects suggesting considerable context sensitivity of the effect (e.g. [10, 11, 12]).

One important moderator addresses the affective-motivational value of the primed concept. Specifically, action-word priming effects seem to be more pronounced when the action-words prime a behavioral concept that has positive value and represents an attractive goal to act on for the individual. Accordingly, primed behavioral concepts that are associated with positive affect (e.g., individual difference or induced in an evaluative conditioning task) are more likely to change corresponding behavior than behavioral concepts that are neutral [13, 14, 15, 16, 17, 18].

Interestingly, there is research to suggest that behavioral concept priming effects are not only facilitated by positive affect. They might also be modulated by negative affect [19, 20, 21, 22]. In a first demonstration of this notion [19], a sample of undergraduate students were exposed to words representing the goal to socialize, i.e. a concept that was assumed to be strongly valued by the participants. They were then given the opportunity to participate in a lottery to earn tickets for a party, but only if enough time was left after performing an intermediate mouse-click task. This allowed speed to be taken as a dependent measure for goal pursuit (wanting to win a ticket to a party). In line with the general finding in the recent meta-analysis [10], the word priming facilitated behavior. Importantly, the effects vanished when the initially valued concept of socializing was co-activated with negative affect. These effects may have important implications for the understanding and examination of word priming effects on behavior; they suggest that action concepts that are valued are more likely to produce behavioral effects, but that these effects cease when the study set-up allows the valued action-concept to be surrounded by negative affect. Because the evidence for this cessation effect is rather scarce, we deemed it important to replicate the basic effect with a larger sample.

Research also revealed the importance of individual differences in moderating behavioral priming effects [12]. Even though this aspect has not been systematically evaluated in the meta-analysis [10], there is collective evidence that individual preferences amplify priming effects when prime words are in line with these preferences [15, 16, 23, 24]. In parallel to the situationally based affective-motivational value of a prime mentioned above, individual preferences can be understood as a heightened chronic affective-motivational value within the person. This heightened value on the part of the person heightens the value of the prime which subsequently leads to stronger priming effects.

In action-word priming research, primes correspond to a specific behavioral domain and the domain-specific content of the primes is supposed to activate the corresponding domain-specific behavior. Rigorously, it should therefore be irrelevant which specific primes are used as long as they correspond to the behavioral domain. For example, primes like “socializing”, “dancing” and “going out” should activate affiliative behavior, irrespective which words are used specifically (the same assumption holds true for domains other than affiliation). To our knowledge, this basic assumption has never been examined. An alternative way of thinking would be that the meaning of specific words with their idiosyncratic meanings beyond their correspondence to a behavioral domain activates psychological processes associated to this specific word.

For example, in the Aarts et al. [19] study, the word “party” was a keyword among the prime words, and the announcement of the lottery task was explicitly framed in terms of “party” as well. Thus, it might be the case that the congruence between the prime word and the word used in the behavioral task matters, and thus effects of primes on behavior might disappear when an alternative word for party is used for the announcement (e.g., when festivity instead of party is used in the announcement). If the alternative announcement wording were equally effective, this would support the notion that the correspondence to the domain of affiliation is activated by the primes, as the word used for the announcement corresponds to the domain of affiliation as well. However, if the effects would not show up for the alternative wording (e.g., festivity instead of party), this would give credit to the alternative explanation that the effect would rest on specific-word associations beyond the correspondence to the domain of affiliation. That action-word priming effects possibly rest on the specific meaning of a word and the close congruence between prime and the announcement of the behavioral task may make priming less reliable. This might explain why some research has failed to replicate behavioral priming effects, and ruling out this alternative explanation is thus of basic interest.

### Present research

In the present study, we focused on a previous study by Aarts et al. [19], which addressed the role of affective conditioning in the social affiliation domain. The first aim was the replication of the cessation effect using the procedure of co-activation of negative affect and affiliative primes reported by Aarts et al. [19]. Second, on the basis of previous findings on the moderation by individual differences, we probed for the moderation by affiliation motive strength, that is, whether the cessation effect is stronger for individuals with a higher affiliation motive (i.e., a higher individual preference). Our third aim was examining whether action-word priming effects on behavior rely on the activation of a broad domain-specific concept rather than specific-word associations.

In our study, we compared effects of a negative conditioning condition (in which participants were exposed to socializing words that were co-activated with negative words) with a neutral conditioning condition (in which participants were exposed to socializing words that were co-activated with neutral words) on the speed of a mouse-click task instrumental for winning tickets for a party. Aarts et al. [19] found a moderately strong effect size in a sample of 75 participants. In general, small samples are noisy in estimating the effect size, and smaller effects are usually expected for replication studies (e.g. [25]). Our estimation of the appropriate sample size for our replication study was based on the recommendation of Simonsohn [26] to include at least 2.5 times the number of participants of the original study to detect small effects [26].

According to our aim to replicate the cessation effect of negative conditioning compared to neutral conditioning, we did not include a control condition with no prime. The comparison of prime and control condition has been intensively studied [10] and in the original study by Aarts et al. [19], the control condition showed similar results as the neutral condition. Leaving out the control condition enhancing the statistical power to detect the effect we aimed for (based on the sample realized, we had nearly four times more subjects for the comparison of neutral vs. negative condition as in the Aarts et al. [19]), further warranted as we realized additional conditions (described below) which potentially added noise to the data.

In order to examine whether action-word priming effects on behavior rely on the activation of a broad domain-specific concept rather than a specific-word associations, we reasoned that synonyms (i.e., words equally representing the concept) should have the same effect (and respectively the same cessation effect). Specifically, Aarts et al. [19] used the word “party” as a prime (along with four other affiliative words) and also as the keyword in the announcement of the lottery task to render the mouse-click task instrumental. If the cessation effect indeed rested on the activation of the domain of affiliation, using a synonym for the word “party” should yield the same result. Thus, we tested whether the cessation effect is stronger when the conditioned prime word is used for announcing the lottery task (congruence condition) compared to when a synonym of this prime word is used for making the announcement (incongruence condition). For this first empirical investigation of our reasoning, we included only one prime word or synonym in the announcement, because we wanted to keep the announcement as similar as possible to the original study [19].

## Materials and method

### Participants

One-hundred and ninety-seven university students participated at the end of an unrelated 90-minute experiment and earned 15€ or course credit. We excluded six participants who responded incorrectly in 50% or more trials in the conditioning task (dot-detection task). For the remaining 191 participants, there was missing data on gender, age, and motive measures for three participants. One subject additionally designated himself/herself as “third gender.” In all these cases, we replaced missing values by the sample means. Participants were *M* = 22.48 (*SD* = 3.22) years of age, and 53 indicated that they were male. The study adhered to the human research protection protocol of Friedrich Schiller University Jena, and was granted and approved by the German Research Foundation. The study was conducted, and written informed consent of each participant was obtained in compliance with the principles contained in the Declaration of Helsinki.

### Design

We created two lists of affiliation primes. One list included all words (translated to German) used in the original study by Aarts et al. [19]), and the other included the same words with the exception of “party”, which was replaced by a (German) synonym (the word “festivity”; The German word for festivity “Feier” is commonly used in natural language.). The study included the neutral conditioning and the negative conditioning condition. We used either the word “party” or its synonym “festivity” to announce the lottery task. Thus we had a 2 (conditioning: neutral vs. negative) x 2 (congruence of prime and announcement: no vs. yes) between-subjects design with the speed in the mouse-click task as the dependent variable. Subjects were randomly assigned to the experimental conditions.

### Procedures and measures

At least one day before the experimental session, demographic information and personality measures for motive dispositions were assessed via Internet. The Unified Motive Scales [27] short version was used to assess the motives for affiliation, intimacy, achievement, power, and fear (6 items per motive; internal consistency *α* > .81) via self-report. Participants were informed that, in the remaining part of the session, several unrelated tasks would be presented. After some general instructions and practice with the computer program, subjects started to work on three consecutive tasks: the goal conditioning task, a mouse-click task and a lottery task.

#### Goal conditioning task

In an adaption of the task used in Aarts et al. [19], subjects performed the dot detection task in which one of two different lists (party list or festivity list) of affiliative primes was associated with either neutral or negative words (see S1 Table). In 50 trials, five affiliation primes and five non-words (random letter strings) were presented five times. In the neutral conditioning condition, each of the affiliation primes was accompanied once by each neutral word, and in the negative conditioning condition, once by each negative word. In order to balance the presentation of neutral and negative words, the remaining half of the trials in the neutral condition nonwords were accompanied with negative words and in the negative conditioning condition non-words were linked to neutral words (thus exposure to negative information is controlled for). Each trial started with a fixation cross (500 ms) followed by a mask of Xs (600 ms), an affiliation prime or a non-word (23 ms), a mask of Xs (200 ms), a neutral or negative word (150 ms), and 23 ms after the word disappeared, in half of the trials, a dot (23 ms; cf. [19]). Stimuli were presented in the middle of the screen and were about 0.50 cm in height. Subjects’ task was to indicate if a dot had appeared by pressing the “j” key, otherwise they should press the “n” key. The next trial started after a pause of 450 ms.

#### Mouse-click task

Subsequently, participants had to perform a mouse-click task in which they had to click on a square (2 x 3 cm) that appeared in different areas of the computer screen. After they clicked on a square, the next square would appear at random locations on the screen (the task was specifically programmed to match the software and set-up, and hence, was different from the one used by Aarts et al. [19]). However, participants also learned that, if enough time was left after the mouse-click task, they could participate in a lottery to win tickets for a student party or student festivity (depending the congruence condition; see wording in S1 Table; wording was very similar to the formulations used in Aarts et al. [19]. Participants were not informed about the number of trials in the mouse-click task. The total amount of time needed for the mouse-click task (35 trials) was the dependent variable.

Finally, participants were informed that they will take part in the lottery task where they could win a ticket for a party followed by an awareness check for the subliminally presented prime words (a word recognition task where they indicated whether a word had been presented in the dot-detection task by pressing yes or no (we presented the affiliation word primes, the five neutral and the five negative words as well as five new nouns not included in the dot-detection task). Finally, participants were debriefed.

## Results

### Awareness check of primed words

In the awareness check (recognition task), participants indicated that they recognized the affiliation word primes in the dot-detection task in 25% of times. This was well below the chance rate of 50% (i.e., pressing randomly yes or no) and was similar to the rate of 28% for new words not included in the dot-detection task. These results indicate that participants were likely unaware of being exposed to the prime words.

### Behavioral effects

**Fig 1:**
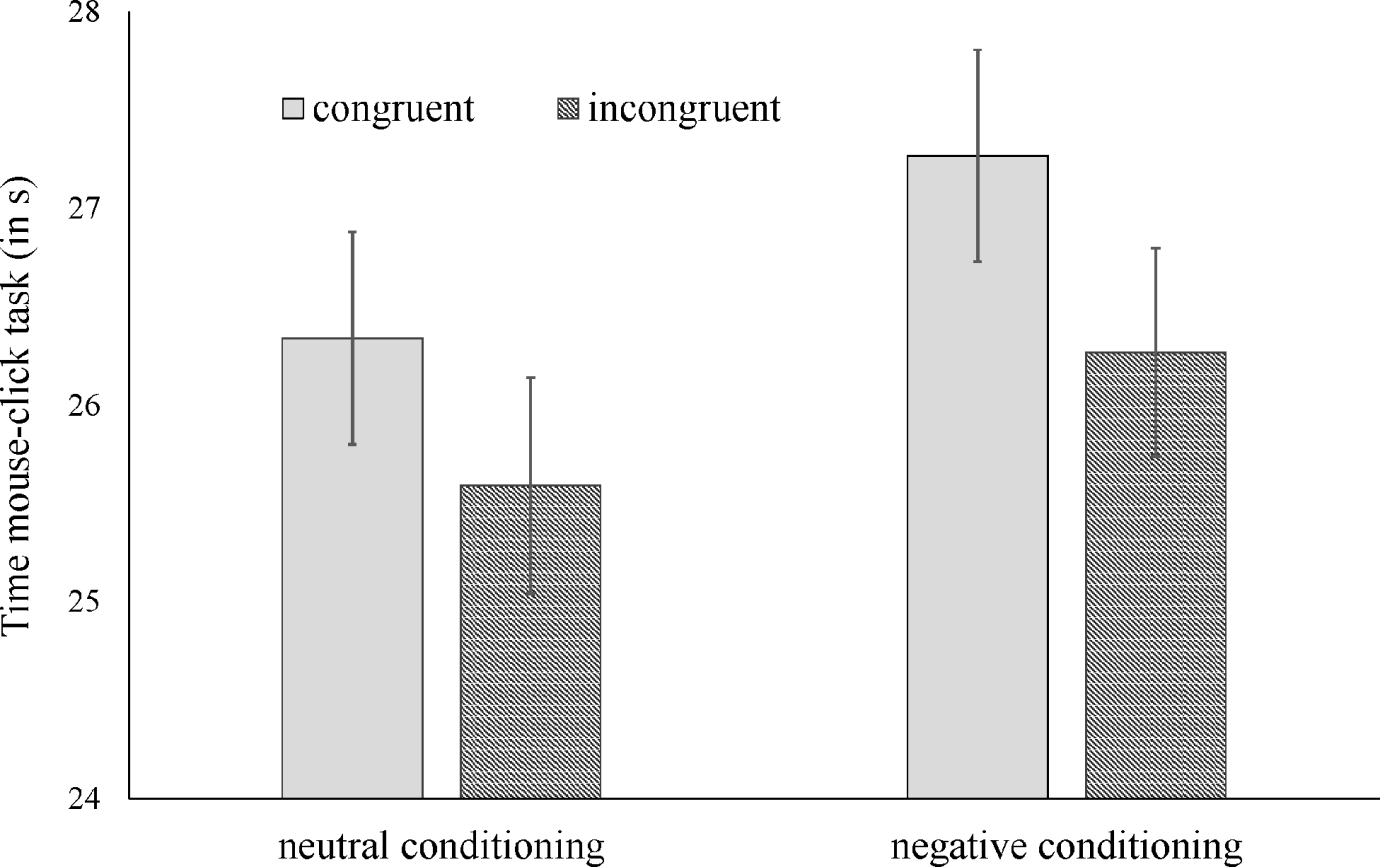
Main and interaction effects of conditioning and congruence on working times in the mouse-click task.

Results of the 2 x 2 factorial ANOVA showed that, in line with the findings reported by Aarts and colleagues [19], participants were faster to complete the mouse-click task in the neutral conditioning condition (*M* = 25.97, *SD* = 3.43) than in the negative conditioning condition (*M* = 26.76, *SD* = 4.04). However, the effect was not significant, *F*(1,187) = 2.20, *p* = .140, *d* = 0.22. Thus, whereas the direction of the cessation effect was compatible with the original finding (cf. Fig 1), the effect seems to be fairly small (and more in line with the effect size obtained in the meta-analysis). Fig 1 also depicts the result for the congruence of prime and announcement. In the congruence condition subjects were faster (i.e., more motivated), but the effect of congruence was not significant, and neither was the interaction effect of conditioning and congruence; *F(*1, 187) = 2.60, *p* = .108, and *F*(1, 187) = 0.05, *p* = .818. This implies that we found no evidence that congruency between word prime and word announcement matters.

#### The moderating role of affiliation motive strength

**Fig 2:**
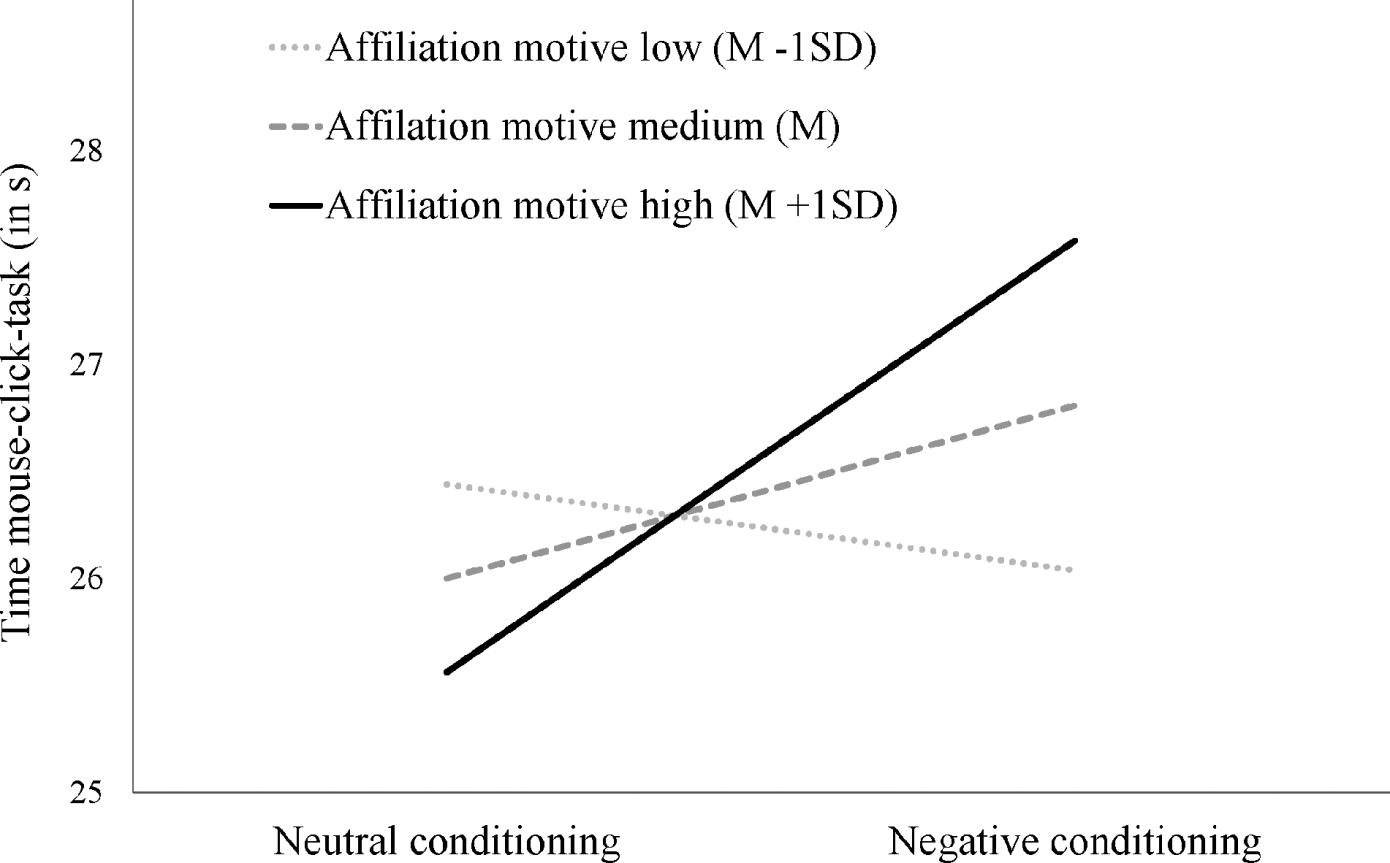
Interaction effects of conditioning and affiliation motive on working times in the mouse-click task.

Because previous research indicates that concept-priming effects on behavior might be conditional on the strength of motivation or value of the primed concept, we tested whether individual differences in the affiliation motive moderated the effect. Accordingly, we conducted an ANCOVA with conditioning condition as factor, the *z*-standardized affiliation motive as covariate and the interaction of both variables. The effect of conditioning was again nonsignificant; *F*(1,187) = 2.25, *p* = .136, *d* = 0.22. The main effect for the affiliation motive was not significant; *F*(1,187) = 0.38, *p* = .540, d = 0.09, *d* = 0.09. However, the interaction effect was significant; *F*(1,187) = 5.00, *p* = .027. The moderation by the affiliation motive is depicted in Fig 2, showing that the cessation effect of affiliative motivation (i.e., longer response times in the negative conditioning condition) was stronger when the affiliation motive was stronger, with the simple slope for a high affiliation motive (1 *SD* above the mean) being moderately strong and significant; *F*(1,187) = 6.97, *p* = .009, *d* = 0.56 (and very weak and not significant for the low affiliation motive [1 *SD* below the mean]; *F*(1,187) = 0.28, *p* = .599, *d* = 0.10). An ANCOVA including congruence as an additional factor revealed neither a significant interaction effect with conditioning nor a three-way interaction with the affiliation motive (*p*s > .388). Thus, there was again no indication that the goal cessation effect rests on the congruence of prime and task word. We also probed for the moderation of intimacy, achievement, power and fear motive. There were no significant main nor interaction effects of these motives; *F*(1,187) < 1.48, *p*s > .224. Thus, the effect exclusively showed up for the topic-specific affiliation motive.

## Discussion

We found that experimentally conditioned negative associations of affiliative action-words led to a cessation of affiliation motivated behavior, but the effect was fairly small and not significant. Despite our large sample size, the main effect of negative conditioning could therefore not be clearly replicated. However, moderation analyses showed that the effect increased with participants’ propensity for greater enjoyment of the company of others (i.e., for participants with higher affiliative motive): For participants with strong affiliation motives the cessation effect was nearly as strong as in the original study [19]. We found no moderation effect of congruence between prime and task word: The cessation effect in participants with strong affiliation motives was independent of whether the central prime word in the conditioning procedure was identical to, or a synonym of the word used in the announcement instruction of the dependent variable. Thus, we found no evidence that the priming effect rests on a specific-word associations beyond the correspondence to the domain of affiliation, and the data are in accord with the assumption that word primes activated the broader concept of affiliation.

Although a clear replication of the original motivation-cessation effect reported by Aarts et al. [19] was not successful, we are hesitant to reject the hypothesis of motivation cessation by negative conditioning altogether. First, the direction of the main effect appeared to be similar to the original findings. In addition, while small, the effect size (*d* = .22) approached the effect size (*d* = .33) found in the meta-analysis by Weingarten et al. [10]. More importantly, however, our study showed that the expected cessation effect was present in participants with strong affiliation motives. This moderating effect of the affiliation motive was specific, as no other motive showed significant interaction effects. Previous research within the affective conditioning paradigm has shown that priming effects are prone to moderation by individual differences in motivation [14, 16, 18, 23, 24], and the same seems to be true for effects of behavioral priming in general (cf. [10, 12]).

Individual differences in motive dispositions can be seen as preexisting differences in the sensitivity for motive-congruent situational cues. It is plausible that primes can unfold their influence only if the prime content is relevant for the individual. Specifically, in the realm of affiliation one may have a friendly social interaction or one may be rejected. Both experiences are more relevant for individuals scoring high on the affiliation motive [28]. In the present case, the prime words related to socializing first motivated participants scoring high in affiliation to socialize. For participants who have no or a weak affiliation motive, however, the prime words might not have triggered the motivation to socialize to begin with. Hence, there was no situationally induced motivation that could be reduced (or ceased) by the negative conditioning treatment (as revealed by no differences between the neutral and the negative conditioning prime condition). Future studies may include a neutral condition without primes. The comparison of the negative vs. no prime condition would provide further information about whether subjects with a high affiliation motive are indeed more sensitive to negative conditioning.

Research suggests that cultural differences are associated with differences in the strength of motivational orientations [29, 30]. It is therefore possible that the Dutch participants in the original study were on average more strongly motivated to socialize and had a higher affiliation motive than the present sample of German participants. However, although this rationale might explain the differences between our results and the findings of Aarts et al. [19], it remains speculative and calls for subjecting such comparison to future studies. Another reason why replication of the main cessation effect in a different sample was not successful may be due to subtle change of meanings in translating the original primes. The translated German primes may not have the same strong affiliative associations as the original primes, but just have a strong affiliative association for sensitive subjects indicated by high affiliation motives.

Finally, our finding of a non-significant difference when using a conditioned prime or a synonym to introduce the lottery does not support the alternative explanation that priming effects rest on the idiosyncratic meaning of prime words. This result therefore does not provide evidence against the basic assumption of action-word priming research that primes can activate a broader concept of affiliation. It also implies, that it is sufficient that announcement of the task solely refer to this broader concept. Our design mirrored Aarts et al. [19] in order to provide a close replication and we therefore refrained from using a design that would test the assumption with even more statistical power. Higher power could be realized by using a second list where most or all primes are synonyms of the first list, and using more than one conditioned prime or synonym for the announcement. Thus, our findings represent a first test, but future studies should further challenge the assumption of concept activation by behavioral priming in order to provide a more conclusive evaluation of this basicassumption. These studies may also include an announcement without any primes presented before in order to reveal the effect of this announcement compared to the congruent or incongruent condition.

## Conclusion

We could replicate the cessation effect for affiliation motivation reported by Aarts et al. [19] when taking individual differences into account. In addition, we found no indication that the priming effect rests on the idiosyncratic meaning of single words, which is in accord with the basic assumption that primes activate the concept of the corresponding behavioral domain. We hope that the present study provides further insights regarding the potential role of affect and motivation in behavioral priming effects, and the debate about replication efforts.

## Supporting information

S1 Table Stimuli used in the experiment (participants were presented the German words). (DOCX)

S1 File Supporting information. (CSV)
