# Supplementary material for: The nonconscious cessation of affiliative motivation: A replication and extension study
Source: PLoS One. 2018 Jun 28;13(6):e0198899. doi: 10.1371/journal.pone.0198899 (PMC6023142; doi:10.1371/journal.pone.0198899)
Supplement: S1 Table — (DOCX) [file pone.0198899.s001.docx]

S1 Table.

| Affiliative Primes |  | Words used for conditioning | |
| --- | --- | --- | --- |
| List 1 / List 2 |  | Neutral-conditioning | Negative-conditioning |
| socializing (sich-treffen)  going out (ausgehen)  party/festivity (Party/Feier)  celebrate (amüsieren)  dancing (tanzen) |  | ballpoint (Kugelschreiber)  bucket (Eimer)  entrance (Eingang)  window (Fenster)  sidewalk (Gehweg) | pain (Schmerz)  disease (Krankheit)  trash (Müll)  sorrow (Leid)  thief (Dieb) |
| Announcement of the lottery (Primes List 1/Primes List 2)  We are almost at the end of this part of the research. What follows is one more part, a mouse-click task. If there is enough time left, you can participate in a lottery where you can win tickets to a student party/festivity in Jena. (German: Nun sind wir so gut wie am Ende von diesem Teil der Untersuchungen. Was jetzt folgt, ist ein weiterer Teil, eine Maus-Klick-Aufgabe. Sollte dann noch genügend Zeit übrig sein, kannst Du an einer Verlosung teilnehmen, bei der Du Tickets für eine Studentenparty/Studentenfeier in Jena gewinnen kannst.) | | | |
